# Eu-Social Science: The Role of Internet Social Networks in the Collection of Bee Biodiversity Data

**DOI:** 10.1371/journal.pone.0014381

**Published:** 2010-12-17

**Authors:** Richard Stafford, Adam G. Hart, Laura Collins, Claire L. Kirkhope, Rachel L. Williams, Samuel G. Rees, Jane R. Lloyd, Anne E. Goodenough

**Affiliations:** Department of Natural and Social Sciences, University of Gloucestershire, Cheltenham, United Kingdom; Smithsonian's National Zoological Park, United States of America

## Abstract

**Background:**

Monitoring change in species diversity, community composition and phenology is vital to assess the impacts of anthropogenic activity and natural change. However, monitoring by trained scientists is time consuming and expensive.

**Methodology/Principal Findings:**

Using social networks, we assess whether it is possible to obtain accurate data on bee distribution across the UK from photographic records submitted by untrained members of the public, and if these data are in sufficient quantity for ecological studies. We used Flickr and Facebook as social networks and Flickr for the storage of photographs and associated data on date, time and location linked to them. Within six weeks, the number of pictures uploaded to the Flickr BeeID group exceeded 200. Geographic coverage was excellent; the distribution of photographs covered most of the British Isles, from the south coast of England to the Highlands of Scotland. However, only 59% of photographs were properly uploaded according to instructions, with vital information such as ‘tags’ or location information missing from the remainder. Nevertheless, this incorporation of information on location of photographs was much higher than general usage on Flickr (∼13%), indicating the need for dedicated projects to collect spatial ecological data. Furthermore, we found identification of bees is not possible from all photographs, especially those excluding lower abdomen detail. This suggests that giving details regarding specific anatomical features to include on photographs would be useful to maximise success.

**Conclusions/Significance:**

The study demonstrates the power of social network sites to generate public interest in a project and details the advantages of using a group within an existing popular social network site over a traditional (specifically-designed) web-based or paper-based submission process. Some advantages include the ability to network with other individuals or groups with similar interests, and thus increasing the size of the dataset and participation in the project.

## Introduction

Citizen science involves volunteers collecting and reporting data for scientists to analyse in subsequent studies [1]. This has many potential benefits. For example, it allows citizens to be actively involved with the natural world and enhances their education [2], while data collection can occur potentially on a global scale, and provide more comprehensive and rapid coverage, than is possible with a team of scientific researchers [3]. Data can also be collected rapidly and cheaply, although there are also potential problems with these methods of data collection [4] (see below).

Many citizen science projects exist and thousands of people are participating in these projects globally. For example, in the UK the National Biodiversity Network now has over 31 million records of plant and animal species largely submitted by amateur naturalists [1]. While some projects have localised scope (for example, UK country-specific bird reports such as that produced in Gloucestershire [5]), others span a wide geographical range. For example, in Australia there are large-scale citizen science projects mapping distributions of species as diverse as possums, whale sharks and frogs [3]. International schemes are also in place; a good example is the EURING bird ringing and recovery scheme that operates across over 30 European countries.

In the past decade, the internet has provided a key advance for citizen science projects, allowing data to be directly entered by users and eliminating the costs and effort associated with paper-based data entry [1]. The development of Web 2.0– or websites that interact with the user – particularly the development of social networks where comments or photographs can be shared with an online community – has many benefits for citizen science data collection. Many citizen science projects therefore have incorporated a social network element or are based solely within social network sites (Table 1).

**Table 1:** Examples of both general, and bee related, web-based citizen science or biodiversity sites. A brief description of the projects is given, as are details regarding of the use of social networks data collection.

Name	Website	Type of Project	Main online presence	Links to social network sites^1^	Link (or twitter tags)
OPAL	http://www.opalexplorenature.org	Citizen science data collection	Interactive Web-based	None	
iSpot^2^	www.ispot.org	UK biodiversity identification	Self-contained Social network	None	
Encyclopaedia of Life	http://www.eol.org/	Web based, wiki style encyclopaedia for biology	Website	Flickr group use to collect images for main project	http://www.flickr.com/groups/encyclopedia_of_life
Great Blue Heron	http://www.flickr.com/groups/csgreatblueheron	Citizen science data and distribution	Flickr Based Group	Flickr based	http://www.flickr.com/groups/csgreatblueheron
BBC Springwatch/Autumnwatch	http://www.bbc.co.uk/nature/uk/	Public entertainment and education	Cooperate website	Flickr Based Group^3^ Twitter messaging	http://www.flickr.com/groups/bbcspringwatch@bbc_autumnwatch @bbc_springwatch
BBC Bee Part of It	http://www.bbc.co.uk/breathingplaces/beepartofit/	Education and conservation	Cooperate website	Flickr Based Group^3^	http://www.flickr.com/groups/bbc_beepartofit/
Great Sunflower Project	http://www.greatsunflower.org/	Citizen Science Bee identification	Group website	Photographs on Flickr link to traditional web-based data submission	http://www.flickr.com/groups/greatsunflower/
Bee Spotter	http://beespotter.mste.illinois.edu/	Citizen science bee identification through photographs	University website	None^4^	

1 Links to key social network sites where information is collected or disseminated are given. Simple ‘fan’ pages on social networks such as Facebook, which just link to other sites are not included.

2 iSpot is a social network component of OPAL.

3 The Flickr site is a collection of photographs of bees, and is not related to the main project aims of setting up bee colonies.

4 Links to many social networks for the purposes of disseminating the project, through individual participants status updates, are given.

Distribution data for a particular taxonomic group (e.g. birds or butterflies) can normally be collected easily through volunteers, but identification problems can make collecting species level taxonomic data difficult for those projects which appeal to the general public (i.e. crowd sourcing projects, rather than data collected by participants with a specific interest in a particular group) [6]. Collecting accurate population size data can also be difficult because of the aggregated nature of data collection and unequal effort between individuals [4]. For behavioural studies, collecting data using different observers has also indicated problems of bias; for example men and women can differ in objective decisions relating to animal behaviour [7], [8].

With greater uptake in new methods of data analysis, such as Bayesian networks that can assign different priors as levels of confidence for the accuracy of the data [9], many of the problems of bias can be overcome, for example, incorrect identifications of species in spurious locations, outside of the normal range, can be detected and accounted for (R. Stafford and J. R. Lloyd, unpublished data; see also discussion below regarding quantification of effort). However, the issues of volunteer motivation (or crowd sourcing) and accuracy of results (in terms of location, species identification etc.) still need to be addressed.

In this study we examine the BeeID project, a citizen science project that maps the distribution of bees throughout the UK. This project attempts to eliminate many of the problems of ‘citizen’ collected data through the use of new technologies such as smartphones. It is based around the use of social network sites, potentially broadening interest and increasing the number of participants. This study compares the success of participation in the project, the scientific validity of the data collected, and the benefits of using social networks for this type of research, with other data collection techniques.

## Methods

The BeeID project was run through the Flickr photosharing website (www.flickr.com). Flickr is a web 2.0 application that allows users to upload their photographs and videos to their server, as well as allowing discussion threads and comments on photographs posted. The BeeID project was set up as a special interest group at in order to keep the project focussed and discrete. The photographs and other discussion material are available to view at (www.flickr.com/groups/beeid).

To attract potential users to the Flickr group, a publicity-oriented Facebook group was set up (the number of users of Facebook vastly exceeds those of other social network sites [10]). Facebook was not used as the main photograph upload site since, although it allows photographs to be uploaded, it removes much of the useful information attached to digital photographs in the Exchangeable Image File format (EXIF) for privacy reasons.

The Flickr group contained instructions for participants. Photographs were requested to be uploaded, added to the BeeID group, and given the unique tag ‘BEEID2010’. Participants were also asked to add their photographs to the Flickr map, either manually, or automatically using the GPS data incorporated in their photograph's EXIF information if GPS was present on the camera or smartphone with which the photograph had be captured.

Images were searched by a computer program written in Python 2.3, which searched for the BEEID2010 tag (see supplementary material Text S1 for the code, which is released under the GNU GPL). The program was capable of extracting the date and time information from the EXIF information (as recorded by the camera) directly, as well as GPS coordinates if present in the EXIF information or on ‘geotagged’ photographs (those with location information added as a machine tag or through the Flickr map). The program used the Python Flickr API software written by James Clark (http://stuvel.eu/projects/flickrapi) as a basis of the interface with the Flickr Application Programming Interface (API).

Images were identified by a team of faculty staff, research students and recent graduates from the Biosciences degree programmes at the University of Gloucestershire. Photographs not readily identifiable were marked as such, and then presented to a team of experts. Photographs were identified to species level where possible (see Table 2 for a list of species/genera identified by the project). A short comment, thanking the contributor for the contribution, and a further tag for the photograph, based on the identification, was given (see Table 2 for tag information).

**Table 2:** The number of each species of bee uploaded, correctly tagged and located on the Flickr map (through geotagging or incorporated GPS data) until 30^th^ June 2010.

Common name	Scientific name	Number correctly tagged	Number correctly ‘geotagged’	Number with GPS data	Processed tag used to search Flickr map
Buff tailed bumblebee	*Bombus terrestris*	22	21	2	processbeeid2010_buff_tail
White Tailed bumblebee	*Bombus lucorum*	3	2	0	processbeeid2010_white_tail
Early Bumblebee	*Bombus pratorum*	20	13	1	processbeeid2010_early_bb
Common carder bee	*Bombus pascuorum*	18	13	0	processbeeid2010_common_carder
Red Tailed Bumblebee	*Bombus lapidarius*	7	7	2	processbeeid2010_red_tail
Bumblebee – not to species	*Bombus* spp.	38	32	4	processbeeid2010_bumblebee_no_id
Honeybee	*Apis mellifera*	14	12	2	processbeeid2010_apis
Mining bee	*Andrena* spp.	13	8	1	processbeeid2010_Andrena
Red mason bee	*Osmia rufa*	8	6	0	processbeeid2010_red_mason
Hairy footed flower bee	*Anthophora* spp.	3	1	0	processbeeid2010_hairy_footed_flower
Mining bee	*Lasioglossum* spp.	2	1	0	processbeeid2010_Lasioglossum
Nomad bee	*Nomada* spp.	4	3	0	processbeeid2010_nomad
Other (non bees)		4	2	0	

A processed photograph was tagged with the initial part of the tag reading ‘processbeeid2010’ (see Table 2 for full tags) and such photographs were ignored in subsequent runs of the program to ensure that only newly-submitted photographs were highlighted for action.

Publicity for the project was initially only through social networking sites (Flickr and Facebook) and included posts on other similar discussion boards. During mid-June 2010, the project was disseminated at the Cheltenham Science Festival, through a free public display in the discovery zone.

## Results

The BeeID project was officially launched on the 11^th^ April 2010. Initially it was promoted solely through Facebook and Flickr groups and obtained 10 contributing members for the Flickr group, but 86 members for the Facebook group. With the promotion of the BBC's Springwatch and BeePartOfIt Flickr sites, and through messages agreed by the group moderators on these groups' Flickr sites, the number of members of the Flickr site increased from 10 to 23 members within 4 days of the posting (posted on the 16^th^ May 2010). As of the end of 23^rd^ June 2010, after promotion at the Cheltenham Science Festival (during the period of 9^th^ –13^th^ June) and promotional work at a “social network” night (Cheltenham Social Media Café), there are 36 members and 206 photographs of bees in the BeeID group pool (equivalent to 4.8 photographs added per day).

**Figure 1:**
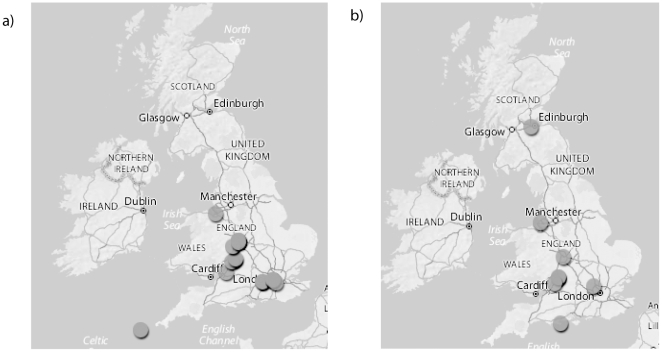
Distribution patterns of species of bees generated from searching by tag (see **Table 1** for tags) using the Flickr map. (a) Distribution of the buff tailed bumblebee (*Bombus terrestris*) – showing similar patterns to those previously reported (i.e. scarce is Scotland). (b) Distribution of the buff tailed bumblebee (*Bombus lapidarius*) indicating its coverage over a wide latitude, even though only 7 photographs were added to the Flickr map. In this case, both the southern and northern most pictures had GPS information attached to the photograph, indicating a high confidence of it being found throughout this range.

Of these photographs, 149 were placed on the Flickr map, and 156 photographs were correctly tagged and found by the Python API programme (some photographs were therefore correctly tagged but not on the map, and some on the map but not correctly tagged). Distributions ranged from the Isles of Scilly in the west, to Lowestoft in the east (the full longitude of the UK), and from Scilly to Glencoe in Scotland in terms of latitude. In total, 11 species were identified from the 156 photographs correctly tagged (numbers of each species are given in Table 2). Bees could not always be identified to species level from these submitted photographs (some species of solitary bee were recorded to genus level only for simplicity – see Table 2). However, there was a particular problem for full identification of photographs of bumblebees, with 35% of uploaded photographs of bumblebees only being identified to genus level. Example distribution patterns obtained for given species are displayed in Figure 1. In total, the number of photographs correctly processed by the public (i.e. both tagged and added to the map) was 121; 59% of the total photographs received. Only 12 photographs (7.7% of the 156 correctly tagged images) had GPS data in the EXIF information, and these were all taken on mobile smartphones.

## Discussion

### Comparison with other citizen science projects

Given that data collection only ran for a short time (10 weeks), there was no funding for this project, the project had no association with any established taxonomic data collection scheme, and that promotion was initially solely through social network sites, the amount of data generated was relatively large. While not a direct comparison for a national project, many regional databases have very few records. For example, calls for members of the public to report Amphibian and Reptile sightings across Gloucestershire in 2008 as part of an annual countywide recording scheme resulted in only 22 sightings of slow worms (*Anguis fragilis*) being submitted; with slow worms being the highest-reported species [11]. Even charismatic species such as basking sharks (*Cetorhinus maximus*), where sightings are both relatively common on the UK coast, but also perceived to be rare and exciting enough to be ‘newsworthy’ and reportable, have relatively low numbers of reported sightings. A national survey run by a well-established conservation group (the Marine Conservation Society) only received ∼10,000 records over 20 years [12].

In comparison with other internet-based ecology or taxonomic projects, the amount of data collected by BeeID was significantly higher than iSpot (www.iSpot.org.uk) during its first year of operation (summer 2009), where only a few photographs were added each day for all taxa covered (mammals, birds, amphibians and reptiles, fish, fungi, lichens and plants). Given the low initial contribution (which, however, was significantly reversed in 2010 by significant funding, prominent links on the BBC's nature website and promotional leaflets available at many wildlife sites throughout the UK, with >50 photographs of insects currently being uploaded per day as of June 2010), this suggests that the use of existing and well established social networking sites have considerable power in increasing participation in citizen science projects. Indeed, large amounts of data generated by the social network approach, could mirror the success of other campaigns, such as political campaigns, conducted via social network sites [10].

The BeeID project received over 200 photographs in the period of operation between April and June 2010. Although this is significantly lower than other similar (but better publicised and longer running projects) such as the BBC's Bee Part of It campaign (with a little under 2,000 photographs as of November 2010), the percentage of photographic submissions to BeeID that contained spatial information (either from EXIF information or from location on the Flickr map) was far higher than for Be Part Of It. Only 25% of photographs from the Be Part of It campaign, as compared to 59% in the BeeID project, had geographical information – despite a request for this to be included in the guidelines. The 59% of BeeID photographs containing spatial data was much higher than general Flickr usage. A search for the tag ‘bee’ produced 393,913 photographs, with only 53,043 (or 13%) containing any sort of location information. This clearly indicates the use of a formal group with clear aims and instructions, but within the framework of an existing social network site, can enhance the collection of scientific data over less formal approaches within social network sites that use images submitted ad-hoc, rather than as part of a specific project.

The ability to use social networking techniques within Flickr – in terms of data collection by group administrators (i.e. posting requests for photographs on discussion forums of other groups) or in terms of the contributors being able to add multiple tags to photographs or submit the same photograph to multiple groups – is a clear method of increasing participation in a project and indicates a clear advantage over developing a specific (non-networking-enabled) data collection site for a new project. Essentially, ease of use for participants is key to success, and indicates why it can be advantageous to use a social network site to collect data directly, rather than a remote website that links to a social network site. In the current study, over 30% of photographs submitted to the BeeID pool were also part of the BBC's Be Part of It campaign, with contributors uploading photographs or adding appropriate tags to already uploaded photographs after a forum post on the Bee Part of It Flickr site. This clearly indicates the use of social networking to increase participation in the project. Other citizen science projects based solely on Flickr use similar techniques. The Great Blue Heron project (see Table 1 for details) asks its members to search for other photographs of the birds on Flickr in general and post a comment asking the contributor of the photograph to submit the photograph to the Great Blue Heron group and include additional information if required.

### Accuracy and limitations of data

Within the BeeID project, slightly over 40% of photographs submitted were not correctly uploaded – not following the instructions precisely or not containing the required information (especially not including geographic information). However, it was easy to exclude these photographs from subsequent analysis using the ‘search by tag’ function of the Flickr API, and using the Flickr map to generate distribution patterns. These processes can be used to eliminate photographs with ‘negligent’ mis-reporting of data. Crucially, the fact that in this project, crowd sourced citizen scientists were only involved in taking the photographs, and not identification, also avoided mistaken identification [1], [6], such that the resultant data were scientifically much more robust than other large scale participation or crowd sourcing projects. However, it must be noted, that this method of increasing accuracy may not be important in many citizen science projects, especially those in which data are generally provided by volunteers with many years of expertise in identification (i.e. experienced amateur naturalists). In fact, identification by such ‘expert’ volunteers may well be more accurate than by practising scientists – especially when dealing with a secondary source of identification – for example from a photograph.

It is also important to note that while ‘negligent’ mis-reporting of data was avoided, wilful mis-reporting was also reduced by the current study. By being able to obtain information on the date the photograph was taken from EXIF information, we could be sure that the majority of photographs were taken during 2010. It is possible to alter the EXIF information of a photograph, but this is a relatively complex and time-consuming task, which is likely to deter most potential data saboteurs. The most recommended program for this on internet forums is ExifTool (http://www.sno.phy.queensu.ca/~phil/exiftool), which operates with a command line interface, and thus is not user friendly.

The requirement of participants to send in photographs of bees resulted in the collection of presence data for a particular species, but not of absence data (i.e. it is unknown if a species is absent from a location or if it is present, but no data has been submitted). Indeed, most photographs are likely to come from areas of, or areas close to, high human populations, where as many bees may be found away from such areas. Areas where bees are not reported could thus be because of a real absence or simply a lack of sampling in these areas [13].

A project such as this, that only requests presence data, can never fully eliminate these problems of sample bias relating to presence-only data. However, the potential ability of social networks to increase the number of participants can at least begin to reduce uncertainty. Where large numbers of volunteers in a given area have submitted presence data for some species, but no data on presence of other species, confidence can be increased that the lack of data on the absent species is due to the true absence of the species, rather than from a lack of sampling effort. While sampling by participants in such a project as this will never be randomised, balanced and fully independent, as required in a well designed scientific survey or experiment (e.g. [14], [15]) the number of photographs submitted from a given location can easily act as a proxy measure for sampling effort, effectively allowing statistical corrections for estimates of diversity to be applied if required [16]–[18].

Given that a crowd sourcing project such as this could result in the collection of long-term data sets, that could be easily used to study changes in the distribution of species over time, common approaches to analysing presence-only data such as that of the ‘climate envelope’ – assuming that a species will exist in areas where climate, or habitat conditions are similar – would be wholly disadvantageous [16]. Even unmodified presence-only data would be able to indicate an extension or contraction of a species' range, as long as a sufficient number of photographs (or effort) had been submitted from a wide geographical area in all years during which the study was operational. However, for such a process to be able to occur, the number of submitted photographs for a study on range distribution would need to be much higher than in the present study. For example, to be sure that a relatively common species, such as the buff tailed bumblebee was changing range or density within an area, an absence in an area covering two or three standard counties of the UK (∼10,000 km^2^) should be determinable from around 30 to 50 submitted photographs of bees from such a region – where other common species were all recorded by photograph. However, for rarer species, a reduction in geographic range or density of a population would be very difficult to determine even if there were 500+ photographs submitted yearly over this area.

Clearly, required numbers of photographs such as those given above do not allow the full exploitation and examination of such data. Analysis techniques such as tracking submission year on year by the username of a contributor who frequently uploaded photographs of rare species would greatly increase the power of the analysis, essentially allowing a ‘repeated measures’ type of analysis to be performed. Indeed, the development of sophisticated analysis techniques that could be used to carefully examine data such as this could potentially be very large, and be very cross disciplinary in nature, clearly spanning the natural sciences (in terms of species distributions) and social sciences (in terms of participant motivation and input).

The current study provides a user-friendly, cheap and effective way to collect biodiversity data for any taxon that can be easily identified from photographs. Moreover, with the increases in demand for the latest smartphones (with higher resolution cameras and better GPS facilities), it is likely to be possible to collect higher numbers of better quality photographs containing GPS data in the EXIF information in the future [19], [20] to further ensure the accuracy of the information obtained.

It is clear from the results of this study that full identification to species level can be difficult from some photographs, even with the well-characterised species studied here. This was especially true for the buff tailed bumblebee (*Bombus terrestris*) and the white-tailed bumblebee (*B. lucorum*) where the main distinguishing feature is in the end of the abdomen, which was not clearly visible in many photographs. While a better definition of photographic protocol (to include abdomen detail) would be useful, it can be difficult to capture this detail photographically, and such a protocol may reduce the number of images submitted. As such, there are potential limitations (as well as the benefits outlined above), in not getting participants to directly identify bees to species level, since this identification would be easier if the the actual insect was seen.

### Recommendations and further work

There are currently a large number of social networks, which could be used for the collection of ecological data. These range from dedicated, specialist self-contained applications such as iSpot, through the development of specialist websites that can link to social network sites to obtain information and images, to the general collection of data from social networks based on what has been uploaded, rather than through specialist groups or using any form of instructions to participants. Advantages and disadvantages of these approaches are given in Table 3. However, we suggest the best approach, especially if funds and time are limited, is the use of a specialist group within an existing social network. The potential of establishing a group within an existing social network for the collection of scientific data is large. Use of social networking sites both facilitates participation in projects, and reduces or eliminates the costs of storing the photographic records on specialised databases. Furthermore, social networks engage the participants in citizen science projects, allowing them to keep track of the project in real time, essential for continued success [1], [21]. Currently, the use of photosharing social network sites (e.g. Flickr or Picasa) appears to be the most useful. Although number of users of sites such as Facebook are much larger, EXIF information is removed from the photograph by the website on upload. Sites such as Twitter could also be useful for citizen science projects, where photographs including key EXIF information such as time, date and location can be uploaded, and it may be possible for participants to ‘follow’ activity of a certain species, or contributor, to keep informed on the progress of the project.

**Table 3:** Advantages and disadvantages of different methods for the incorporation of social networks within citizen science projects.

Technique	Example(s)	Web address(es)	Advantages	Disadvantages	Ideal usage
Self-contained social network	iSpot	www.ispot.org	Total control of upload and information collection process	No immediate public presence. No methods to share data directly with similar groups.High cost of set up and publicity*	Long-term and well funded studies
Web portal with links to social networks to collect data*	Great Sunflower Project	http://www.greatsunflower.org/	High level of control of data collection.Use of alternative databases for storing of data such as photographs (reduced cost and enhanced backup)	More than one interface for usersStill a reliance on standard web-based information upload (including possible mistakes).	Where photographs are supplementary to the main data collection process
Self-contained group within existing social network	BeeIDGreat Blue Heron	http://www.flickr.com/groups/beeid http://www.flickr.com/groups/csgreatblueheron	Negligible set up costs.Able to network with similar groups to share data and increase participation.Generally a high degree of conformation with instructions.Contributors can monitor results themselves in real time (i.e. generate distribution maps)	Extraction of data best achieved though interfacing with website's APILimitations of social networks rules and regulationNo (or limited) ability for ‘branding’	Short- to long-term focussed projects where immediate participation is important or where funding for set up and publicity is limited
Data mining of existing social networks	Unknown for biological researchSee [25] for examples	n/a	Instant access to large (if messy) datasets.Geographical spread of images could be very large (world-wide). This could also be a disadvantage if species of interest has limited range.	Diverse types of data, not standardised in terms of information present and of unknown quality/robustness.Most images do not contain information such as location, making mapping opportunities rare.	Speculative research on existing data.

*http://scratchpads.eu/is a resource for developing websites for biodiversity projects with integrated support for connecting to social network APIs and therefore reducing setup time and costs.

### Conclusions

Use of social networks can have many potential uses for collecting scientific data. Not only can these include interactive maps of species distributions, as shown here, be generated, but also, given time and date information in EXIF information, phenology of species could also be studied. Furthermore, given the development of individual recognition techniques for many species such as turtles, cetaceans or other large charismatic marine or terrestrial vertebrates [22]–[24](Arzoumanian et al., 2005; Kitchen-Wheeler, 2010; Lloyd et al., 2010); it may be possible to use similar techniques of social network photosharing to monitor population sizes and measure behaviour and movement of individual animals using citizen scientists' photographs.

In order to facilitate uptake of the technique, we supply the Python source code for searching the Flickr website and extracting data as supplementary material (Text S1). The corresponding author will be happy to advise or make minor changes to this code for other biodiversity or ecology based projects.

## Supporting Information

Text S1 Python code used to interface with the Flickr API and search by tag for unprocessed photographs
(0.02 MB TXT)
